# Beyond the metabolic syndrome: Visceral and marrow adipose tissues impair bone quantity and quality in Cushing’s disease

**DOI:** 10.1371/journal.pone.0223432

**Published:** 2019-10-15

**Authors:** Sérgio Luchini Batista, Iana Mizumukai de Araújo, Adriana Lelis Carvalho, Maria Augusta V. S. D. Alencar, Andressa K. Nahas, Jorge Elias, Marcello H. Nogueira-Barbosa, Carlos E. G. Salmon, Paula C. L. Elias, Ayrton C. Moreira, Margaret Castro, Francisco J. A. de Paula

**Affiliations:** 1 Department of Clinical Medicine, Ribeirão Preto Medical School, USP, Ribeirão Preto, SP, Brazil; 2 Faculty of Public Health, USP, São Paulo, SP, Brazil; 3 Department of Physics, Faculty of Philosophy, Sciences and Letters of Ribeirão Preto, USP, Ribeirão Preto, SP, Brazil; University of Cordoba, SPAIN

## Abstract

The present study was designed to evaluate the relationship between bone traits [bone mineral density (BMD) and trabecular bone score (TBS)] and the accumulation of fat in adipose tissues [abdominal subcutaneous (SAT), visceral (VAT), marrow (MAT) and intrahepatic lipids (IHL)], as well as insulin resistance, in subjects with Cushing’s disease (CD). The study included control (C = 27), paired (P = 16) and Cushing’s disease (CD = 10) groups, which underwent biochemical assessment, dual X-ray absorptiometry, TBS, and magnetic resonance imaging to determine fat deposits. The CD group showed higher serum levels of glucose and insulin, as well as HOMA-IR values, but lower circulatory levels of osteocalcin, in comparison to C and P. The CD group exhibited lower L1-L4 BMD than P (P = 1.059 ± 0.141 vs CD = 0.935 ± 0.093 g/cm^2^, p < 0.05) (Fig 1A). The lumbar spine BMD from the C group was similar to the other groups. TBS was lower in CD than in P and C (C = 1.512±0.077 vs P = 1.405±0.150 vs CD = 1.135±0.136; p<0.05); there was also significant difference between C and P (p<0.05). MAT, VAT, and IHL were higher in CD than in C and P (p<0.05). Considering all subjects, there was a positive association between TBS with both lumbar spine BMD (R^2^ = 0.45; p<0.0001) and osteocalcin (R^2^ = 0.44; p = 0.05). TBS was negatively associated with MAT (R^2^ = 0.49; p = 0.01), VAT (R^2^ = 0.55; p<0.05), and HOMA-IR (R^2^ = 0.44; p<0.01). MAT was positively related with VAT (R^2^ = 0.44; p<0.01) and IHL (R^2^ = 0.41; p<0.05). In CD, insulin resistance and adipose tissue dysfunction, including high MAT, are active players in bone deterioration, as confirmed by lower lumbar spine BMD and lower TBS. Thus, our findings point to an additional component of the already well-known complex mechanisms of osteoporosis associated with hypercortisolism.

## Introduction

Obesity, the most common metabolic disorder in human beings, increases the incidence of diseases with high mortality and morbidity rates, such as diabetes mellitus, cardiovascular disease, and arterial hypertension [1]. On the other hand, obesity has a dual effect on bone; it positively affects bone mass, but likely impairs bone quality, resulting in no protective effect on fracture risk [2]. In Cushing’s disease (CD), an endocrine cause of obesity, the hypercortisolism creates a toxic hormonal and metabolic environment, increasing the incidence of metabolic and cardiovascular diseases [3]. In addition, glucocorticoid excess deteriorates both bone quantity and quality, generating a very high fracture risk at all levels of bone mineral density (BMD) [4, 5]. Endogenous hypercortisolism represents a more appropriate clinical model for investigating the metabolic and skeletal effects of glucocorticoids as compared to exogenous glucocorticoid therapy, since it avoids the confounding effects of an unrelated disease.

Low bone mass detected by dual X-ray energy absorptiometry (DXA) persists as one of the most important indicators of bone fracture susceptibility [6]. However, in glucocorticoid-induced osteoporosis, as well as in obesity and type 2 diabetes mellitus, the concomitant impairment in bone quality hampers the capability of DXA to efficiently capture the risk of fracture [7]. The trabecular bone score (TBS), has been accepted as an option to detect impairment in bone quality in these conditions [8].

Over the last two decades, a complex network linking the modulation of bone remodeling to adipose tissue metabolism has been revealed [9]. The accumulation of fat in adipose tissue, as well as the overflow of lipids into other tissues, creates an inflammatory environment that is a basis for the emergence of severe disorders (e.g., type 2 diabetes mellitus, cardiovascular disorders, and cirrhosis) [10]. There is no consensus in the literature concerning the association of bone phenotype to adipose tissue distribution and insulin resistance. A previous study in obese adolescent girls reported a negative relationship between bone mineral density (BMD) and the rate of visceral adipose tissue (VAT)/subcutaneous adipose tissue (SAT) [11]. However, no associations between BMD and VAT, as well as between BMD and insulin resistance, were found in adult non-diabetic women [12]. Moreover, in individuals diagnosed with type 2 diabetes mellitus, no relationship was observed between VAT and BMD [13]. CD typically stimulates the development of central obesity [14], and previous studies have documented significant muscle deposition of lipids in this condition.[15] The interaction between adipose tissue and the skeleton has an additional ingredient, as special adipocytes within marrow adipose tissue (MAT) are part of the universe of cells composing the bone marrow. MAT, at least in humans, shows crucial adaptive differences in response to nutritional fluctuations by expanding during periods of calorie restriction while appearing not to be a site for fat storage during calorie surplus [13, 16, 17]. Previous studies show that MAT expands in patients with autoimmune diseases submitted to glucocorticoid therapy, as well as in individuals diagnosed with CD [15, 18].

The association of insulin resistance and adipose tissue deposits to bone quality and quantity has scarcely been investigated. Thus, we assessed the spectrum of these parameters in normal weight, paired, and CD individuals in order to better understand the interactions of glucose, lipids, and bone metabolism alterations in this hormonal disease.

## Subjects and methods

### Subjects

The present study was approved by the Institutional Review Board of the University Hospital, Ribeirão Preto Medical School, University of São Paulo (# 26087/2012); all subjects agreed to participate and signed informed consent forms.

The study had a cross-sectional design, comprising age and height matched controls (C = 27), paired individuals (P = 16), and patients diagnosed with CD (n = 10). In addition, the P and CD groups were matched by BMI. All control and paired individuals were women older than 18 years without previous evidence of metabolic bone disease and denied any acute disorder within the last 30 days. Individuals diagnosed with type 2 diabetes mellitus were excluded from the C and P groups. In the CD group, no individual had previous diagnosis of type 2 diabetes mellitus, but 6 developed diabetes mellitus associated with hypercortisolism. These individuals were not excluded from the investigation because type 2 diabetes mellitus does not have a detrimental effect on bone mass. Individuals reporting smoking, alcohol drinking habits, using drugs that affect bone metabolism (e.g., bisphosphonates, teriparatide, estrogen, raloxifene, serotonin reuptake inhibitors, and anticonvulsants), or previously submitted to bariatric surgery were also excluded from the three groups. Subjects with CD diagnosis were attending the Neuroendocrinology Outpatient Clinic at the University Hospital (HC-FMRP-USP). The inclusion criteria of the CD subjects were: female, age range 18–65 years. All of the individuals from the CD group had been submitted to laboratory exams to confirm the etiology of endogenous hypercortisolism. They had been submitted to transphenoidal hypophysectomy, with at least temporary cure of CD. Those showing current clinical and laboratory evidence of autoimmune diseases (including type 1 diabetes mellitus), as well as chronic liver, kidney, or thyroid diseases were excluded. In the CD group, 6 individuals had secondary amenorrhea (hypogonadotrophic hypogonadism) and 2 women had menstrual irregularity. The other two women were in menopause. The diagnosis of endogenous hypercortisolism and ACTH/pituitary-dependent disease was confirmed as previously described [19, 20].

### Methods

Blood samples were obtained between 0800 and 0900 hours, after a 10-hour overnight fast. The biochemical assessment of total calcium, albumin, inorganic phosphorus, alkaline phosphatase, creatinine, and glucose was performed in an automatic biochemical analyzer (CT 600i, Wiener Lab Group, Rosario, Argentina). Serum levels of 25-hydroxyvitamin D (25-OHD) (Liaison, DiaSorin, Saluggia VC, Italy), intact parathyroid hormone (PTH) (Immulite, Siemens, Los Angeles, CA, USA), and insulin-like growth factor 1 (IGF1) (Immulite 2000 Siemens, Los Angeles, CA, USA) were determined by chemiluminescence. Serum Osteocalcin (OC) (hOST-EASIA Diasource, Louvain-la-Neuve, Belgium), leptin (Quidel, TECO medical Group, Gewerbestrasse, Sissach, Switzerland), and adiponectin (Millipore, Billerica, MA, USA) were determined by enzyme immunoassay. The measurements of salivary cortisol were performed via the previously described method [21]. All intra- and inter-assay coefficients of variation were lower than 10% and 20%, respectively. Insulin resistance was estimated using the homeostasis model assessment of insulin resistance (HOMA-IR) with the formula: fasting serum insulin (μUI/ml) × fasting plasma glucose (mmol/l)/22.5 [22].

### Dual-energy X-ray absorptiometry

Bone mineral density in the lumbar spine (L1–L4), total hip, and femoral neck was determined by dual-energy X-ray absorptiometry (Hologic Discovery Wi, QDR series, Waltham, MA, USA). The precision error was 1.2% for L1–L4, 2.3% for the femoral neck, and 2.7% for total hip. BMD values were expressed as g/cm^2^. Trabecular Bone Score (TBS) was evaluated in L1-L4 using the TBS iNsight Software version 2.2 (Medimaps, Geneva, Switzerland).

### ^*1*^H-MR spectroscopy of bone marrow in L3

The volunteers underwent spine MRI in a 1.5 T system (Philips ACHIEVA, Philips Medical Systems), as previously described [12]. In brief, the subjects were positioned head first in the magnet bore in the prone position. A phased-array coil was positioned over the lumbar region. Sagittal T2-weighted fast spin echo acquisition was used as a reference for the spectroscopy voxel placement. A single voxel of 1.5 x 1.5 x 1.5 cm^3^ was positioned at the center of the third lumbar (L3) vertebral body. The point resolved spectroscopy (PRESS) technique was applied using the following parameters: repetition time (TR) = 2000 ms, three echo times (TE) = 40/60/80 ms, 8 averages, without fat or water suppression.

MRS data were processed with the LCModel software (Version 6.1, http://www.s-provencher.com/pages/lcmodel.shtml). The area values of the CH2 lipid peak at 1.3 ppm and the water peak at 4.7 ppm were T2-corrected using a fitting to a mono-exponential decay curve. Finally, the MAT content of L3 was estimated as previously described [23].

### Abdominal magnetic resonance imaging

Abdominal images were acquired with a phased-array torso coil. A coronal turbo-spin-echo (TSE) T2-weighted sequence with breath-holding was applied to localize the following scan volumes. Consecutively, two sets of breath-holding axial gradient double-echo T1-weighted sequences, in phase (echo time = 4.2 ms) and out of phase (echo time = 2.1 ms, slice thickness = 6.0 mm), were acquired, one including the upper abdomen and another centered on the umbilical region.

The formula: fat = (SI in phase − SI out of phase)/(2·SI − in phase) was used to calculate IHL from the averaged signal intensity (SI) in each region of interest (ROI). The SI values in the previously mentioned formula refer to the in/out phase images. In the liver, a manual segmentation was performed to select four ROIs as representative segments of the liver at the level of the main portal vein. The label of the visceral and subcutaneous fat areas was defined using the Display software (http://www.bic.mni.mcgill.ca/software/Display/Display.html) and a semiautomatic segmentation of an axial slice at the level of the umbilicus. This methodology has been previously described in detail elsewhere [12].

### Statistical analysis

The comparison of the results obtained from the 3 groups was performed by simple variance (ANOVA) with a factor (one-way), followed by the Duncan post-test, using the PROC GLM procedure of the SAS version 9.4 software (SAS Institute Inc., SAS/STAT User’s Guide, Version 9.4, Cary, NC, USA: SAS Institute INC., 2013). This model requires that the residuals follow a normal distribution with constant variance. To determine the association between the variables of interest, simple (model 1) and adjusted (by age and BMI, model 2) linear regression models were tested considering all subjects and yielded the regression coefficients and R^2^. The level of significance was set at 0.05.

## Results

The anthropometric characteristics and biochemical evaluation of 27 healthy controls, 16 paired subjects, and 10 CD individuals are exhibited in Table 1. The three groups were matched by age and height. The P and CD groups were matched by BMI, whereas the C group showed lower values. The BMI ranges of the CD and P groups, respectively, were 24.5 to 44.3 kg/m^2^ and 22.9 to 41.4 kg/m^2^. Although, these two groups had similar age and BMI, the CD group showed higher VAT, IHL and MAT than the P group.

**Table 1:** Clinical characteristics of the patients, biochemical measurements, BMD, TBS, SAT, VAT, IHL and MAT results.

	C	P	CD
Age (years)	35.4 ± 8.9	40.0 ± 8.4	36.8 ± 11.5
Height (m)	1.65 ± 0.08	1.59 ± 0.07 #	1.63 ± 0.04
Weight (kg)	61.1 ± 7.8	80.3 ± 17.1 #	90.3 ± 15.2 *
Body mass index (kg/m^2^)	22.6 ± 2.6	31.5 ± 5.2 #	34.0 ± 5.1 *
Time of diagnostic (years)	-	-	3.7 ± 1.6
Glucose (mmol/L)	4.75 ± 0.3	5.0 ± 0.5	7.7 ± 3.5 * §
Insulin (pmol/L)	50 ± 23	99 ± 45	226 ± 171 * §
HOMA-IR	1.56 ± 0.75	3.12 ± 1.41	11.81 ± 8.68 * §
HbA1c (%)	5.2 ± 0.3	5.6 ± 0.4	8.0 ± 1.9 * §
Albumin (g/L)	43 ± 2	42 ± 3	43 ± 2
Total Calcium (mmol/L)	2.4 ± 0.1	2.4 ± 0.1	2.4 ± 0.1
Phosphorus (mmol/L)	1.16 ± 0.12	1.09 ± 0.09	1.22 ± 0.22
Alkaline phosphatase (U/L)	143.8 28.4	174.6 ± 50.2	193.3 ± 45.7 *
Creatinine (μmol/L)	62.0 ± 8.8	70.7 ± 8.8	79.5 ± 17.7
IGF1 (ng/dL)	232.5 ± 85.3	192.3 ± 97.5	213.6 ± 82.6
25(OH)D (ng/mL)	23.8 ± 9.0	22.1 ± 5.6	16.6 ± 7.1 *
PTH (pg/ml)	44.5 ± 21.3	39.6 ± 19.8	30.5 ± 15.8
Osteocalcin (ng/mL)	8.8 ± 3.5	6.2 ± 2.7 #	3.2 ± 1.2 *§
Adiponectin (ng/mL)	21.7 ± 14.8	15.1 ± 18.9	9.0 ± 4.6 *
Leptin (μg/L)	18.6 ± 8.9	50.9 ± 20.2 #	65.5 ± 26.9 *
L1–L4 BMD (g/cm^2^)	1.022 ± 0.101	1.059 ± 0.141	0.935 ± 0.093§
Total hip BMD (g/cm^2^)	0.937 ± 0.093	0.972 ± 0.106	0.960 ± 0.117
Total hip BMD/BMI (g/cm^2^**/** kg/m^2^)	0.042±0.005	0.032±0.006#	0.028±0.002*
Femoral neck BMD (g/cm^2^)	0.831 ± 0.106	0.887 ± 0.156	0.866 ± 0.198
Femoral neck BMD/BMI (g/cm^2^**/** kg/m^2^)	0.037±0.005	0.029±0.008#	0.025±0.003*
TBS	1.512 ± 0.077	1.405 ± 0.150 #	1.135 ± 0.136 * §
SAT (mm^2^)	18168 ± 8186	38769 ± 14877 #	36771 ± 18262 *
VAT (mm^2^)	2393 ± 2313	9569 ± 4095 #	14995 ± 6067 * §
VAT/SAT	0.12 ± 0.07	0.28 ± 0.14 #	0.51 ± 0.37 *
IHL (%)	1.61 ± 0.93	8.59 ± 8.39	17.23 ± 15.62 *§
MAT (%)	23.5 ± 8.6	28.0 ± 6.3	39.6 ± 8.6 * §

Abbreviations: C = control group; P = paired group; CD = Cushing's disease group; HOMA-IR = Homeostatic model assessment—insulin resistance; HbA1c = glycosylated hemoglobin; IGF1 = insulin-like growth factor type I, 25(OH)D = 25-hydroxyvitamin D; PTH = parathyroid hormone; BMD = bone mineral density; TBS = trabecular bone score; SAT = subcutaneous adipose tissue; VAT = visceral adipose tissue, IHL = intra-hepatic lipids; MAT = marrow adipose tissue

The measurements of VAT, SAT, and IHL were performed in 13 subjects from C, 9 subjects from P and 8 subjects from CD group. MAT was estimated in 25 subjects from C, 14 subjects from P and all subjects from CD group.

* Means difference between CD when compared to group C, p<0.05

§ means difference between the CD group when compared to the P group, p<0.05

# Means difference between group P when compared to group C, p<0.05

Glucose and insulin serum levels and HOMA-IR values were significantly higher in the CD group than in the other two, but there was no difference between the C and P groups. The circulatory levels of HbA1c were higher in the CD group than in the C and P groups, but there was no difference between the C and P groups. The serum levels of calcium, phosphorus, and PTH were similar in the three groups, but CD showed lower levels of 25-OHD than C. The serum levels of OC in CD were lower than in C and P; OC was also lower in P than C. There was no difference in the serum levels of adiponectin between groups C (21.7 ± 14.8 ng/mL) and P (15.1 ± 18.9 ng/mL), but the CD individuals (9.0 ± 4.6 ng/mL) showed lower adiponectin levels than C (p<0.05). Both the P (50.9 ± 20.2 μg/L) and CD (65.5 ± 26.9 μg/L) groups showed higher serum levels of leptin than the C (18.6 ± 8.9 μg/L) group (p<0.05); leptin levels were also higher in CD than in P (p<0.05). Basal serum levels of cortisol vary from 7.2 to 33 μg/dL. Salivary levels of cortisol at 9h, 23h, and after 1 mg of dexamethasone in CD group were respectively: 2584±1350; 1716±1118; 2372±2227 ng/dL. These measurements reflect both the abnormal pattern of the circadian rhythm as well as the lack of cortisol suppression in response to a low-dose of dexamethasone exhibited by the CD group.

**Fig 1:**
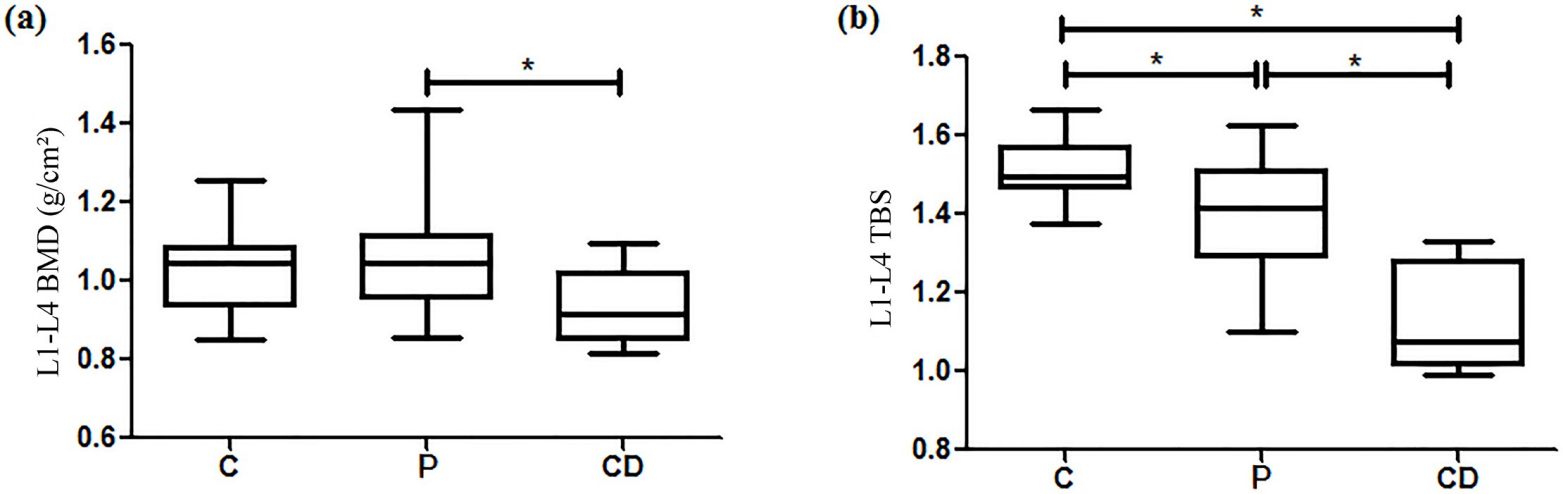
Box-plots of (a) lumbar spine (L1-L4) bone mineral density (BMD) and (b) lumbar spine (L1-L4) trabecular bone score (TBS) in control (C), paired (P) and Cushing’s disease (CD) groups.

The CD group had lower L1-l4 BMD than the P group (P = 1.059 ± 0.141 vs CD = 0.935 ± 0.093 g/cm^2^; p < 0.05) (Fig 1A). The L1-L4 BMD from the C group was similar to the other groups. There was no difference in the BMD in femoral neck and total hip among the three groups. However, the control group showed higher rate of BMD/BMI in total hip (C = 0.042 ± 0.005 vs P = 0.032 ± 0.006 vs CD = 0.028 ± 0.002 g.m^2^/Kg.cm^2;^ p<0.05) and femoral neck (C = 0.0372 ± 0.005 vs P = 0.029 ± 0.008 vs CD = 0.025 ± 0.003 g.m^2^/Kg.cm^2;^ p<0.05) than the other two groups. TBS was lower in CD than in P and C (C = 1.512±0.077 vs P = 1.405±0.150 vs CD = 1.135±0.136; p< 0.05) and there was also significant difference between C and P (p< 0.05) (Fig 1B). As only the CD group had individuals diagnosed with diabetes mellitus, the TBS values in those with and without the associated hyperglycemic disorder were further analyzed. The values of TBS in the CD individuals with diabetes mellitus (1.152±0.166) were similar to those in CD subjects without diabetes mellitus (1.113±0.108).

**Fig 2:**
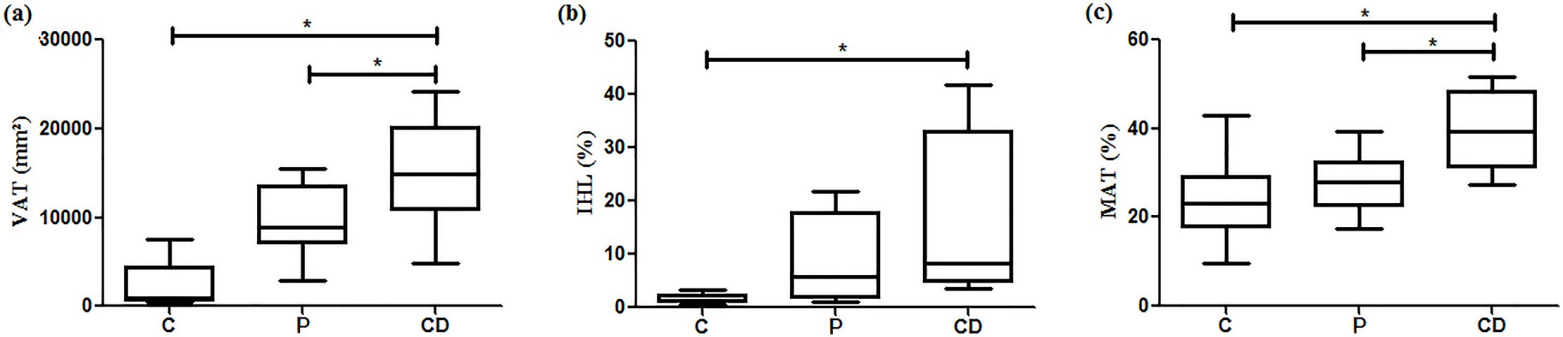
Box-plots of (a) visceral adipose tissue (VAT), (b) intrahepatic lipids (IHL), and (c) marrow adipose tissue (MAT) in control (C), paired (P) and Cushing’s disease (CD) groups.

The measurement of adipose tissue amount and distribution revealed differences among the three groups. The stereotype of unhealthy obesity was strongly associated with the CD group, which showed higher values of VAT than both the C and P groups (C = 2393 ± 2313 vs P = 9569 ± 4095 vs CD = 14995 ± 6067 mm^2^; p<0.05). VAT was higher in P than C (p < 0.05) (Fig 2A). IHL was higher in the CD group in comparison to the two other groups (C = 1.61 ± 0.93 vs P = 8.59 ± 8.39 vs CD = 17.23 ± 15.62%; p < 0.05), whereas there was no difference between the C and P groups (Fig 2B). Moreover, the amount of MAT was significantly higher in CD than in C and P (C = 23.5 ± 8.6 vs P = 28 ± 6.3 vs CD = 39.6 ± 8.6%; p < 0.05), whereas there was no difference between C and P (Fig 2C).

**Fig 3:**
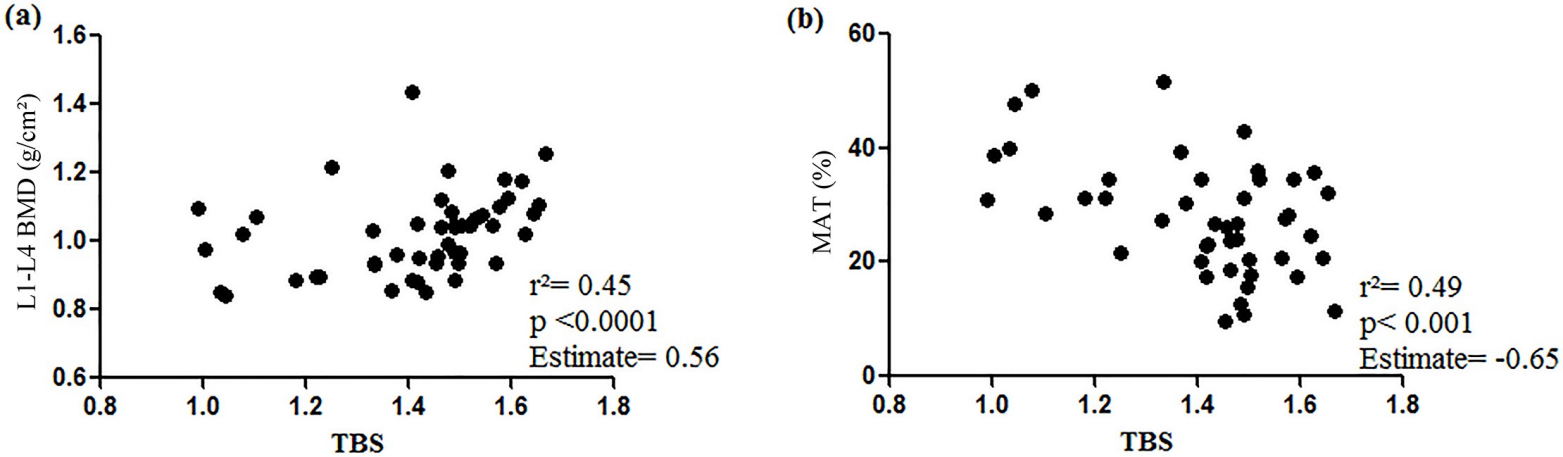
(a) Association between lumbar spine (L1-L4) trabecular bone score (TBS), and lumbar spine (L1-L4) bone mineral density (BMD) and (b) association between lumbar spine (L1-L4) trabecular bone score (TBS) and marrow adipose tissue (MAT).

There was no relationship between basal cortisol with L1-L4 BMD (r = -0.16; p = 0.7), TBS (r = -0.11; p = 0.8) and adiponectin (r = 0.15; p = 0.7) in the CD group. Considering all individuals from the three groups, there was a positive relationship between BMI and MAT (r = 0.32; p < 0.05), VAT (r = 0.76; p < 0.0001), and HOMA-IR (r = 0.53; p < 0.0001) in the 3 groups. There was a positive association between TBS and L1-L4 BMD, even after adjustment for age and weight (R^2^ = 0.45; p<0.0001, estimate = 0.56) (Fig 3A). On the other hand, TBS was negatively associated with MAT (R^2^ = 0.49; p<0.01; estimate = -0.65) (Fig 3B), VAT (R^2^ = 0.55; p < 0.05; estimate = -0.00002), and HOMA-IR (R^2^ = 0.44; p < 0.01; estimate = -0.011), even after adjustment for age and weight (Table 2). TBS was positively associated with OC, after adjustment for age and body weight (R^2^ = 0.44; estimate = 0.013), with a p value equal to 0.05 (Table 2).

**Table 2:** Linear regression analysis.

Associations	Model 1			Model 2*		
	Estimate	p-value	R^2^	Estimate	p-value	R^2^
L1-L4 BMD x TBS	0.230	0.020	0.12	0.562	<0.0001	0.45
L1-L4 BMD x MAT	-0.269	0.130	0.05	-0.444	0.040	0.06
L1-L4 BMD x VAT	0.000	0.740	0.00	0.000	0.520	0.08
L1-L4 BMD x SAT	0.000	0.150	0.07	0.000	0.480	0.00
L1-L4 BMD x IHL	0.002	0.300	0.04	0.001	0.650	0.00
L1-L4 BMD x HOMA-IR	-0.002	0.500	0.01	-0.004	0.260	0.00
L1-L4 BMD x Leptin	0.000	0.820	0.00	-0.001	0.160	0.00
L1-L4 BMD x Adiponectin	0.000	0.700	0.00	0.000	0.900	0.00
L1-L4 BMD x Osteocalcin	-0.004	0.460	0.01	-0.002	0.780	0.00
L1-L4 BMD x IGF1	0.000	0.360	0.02	0.000	0.140	0.01
TBS x MAT	-0.872	0.001	0.23	-0.647	0.008	0.49
TBS x VAT	0.000	<0.0001	0.56	0.000	0.040	0.55
TBS x SAT	0.000	0.0002	0.45	0.000	0.610	0.46
TBS x IHL	-0.011	0.0004	0.42	-0.005	0.100	0.51
TBS x HOMA-IR	-0.018	<0.0001	0.34	-0.011	0.008	0.44
TBS x Osteocalcin	0.026	0.0001	0.28	0.013	0.050	0.44
TBS x IGF1	0.000	0.330	0.02	0.000	0.710	0.40
MAT x TBS	-0.577	0.0008	0.23	-0.604	0.003	0.35
MAT x VAT	0.000	0.0004	0.37	0.000	0.008	0.44
MAT x SAT	0.000	0.390	0.03	0.000	0.140	0.32
MAT x IHL	0.012	0.002	0.29	0.010	0.020	0.41
MAT x HOMA-IR	0.013	0.030	0.11	0.009	0.110	0.33
MAT x Leptin	0.003	0.020	0.11	0.001	0.500	0.24
MAT x Adiponectin	-0.005	0.120	0.07	-0.003	0.300	0.26
MAT x Osteocalcin	-0.024	0.005	0.16	-0.013	0.160	0.26
MAT x IGF1	-0.001	0.100	0.06	0.000	0.390	0.25
VAT x IHL	508009.000	<0.0001	0.70	366267.000	<0.0001	0.79
VAT x HOMA-IR	788391.000	0.0004	0.41	470086.000	0.006	0.71
VAT x Leptin	153497.000	0.0008	0.34	12471.000	0.810	0.53
VAT x Adiponectin	-243765.000	0.004	0.28	-138426.000	0.030	0.61
VAT x Pref1	6133.000	0.810	0.00	5919.000	0.750	0.48
IHL x HOMA-IR	0.991	0.008	0.26	0.666	0.090	0.30
IHL x Leptin	0.157	0.050	0.13	-0.058	0.590	0.28
IHL x Adiponectin	-0.309	0.030	0.17	-0.175	0.180	0.32
IHL x Pref1	0.032	0.480	0.03	0.032	0.400	0.25
IHL x IGF1	-0.033	0.210	0.06	-0.017	0.560	0.28

Notice.

* Model 2 adjusted by age and weight.

L1-L4 BMD = lumbar spine bone mineral density; HOMA-IR = Homeostatic model assessment—insulin resistance; HbA1c = glycosylated hemoglobin; IGF-I = insulin-like growth factor type I, 25(OH)D = 25-hydroxyvitamin D; PTH = parathyroid hormone; Pref-1 = preadipocyte factor; TBS = trabecular bone score; SAT = subcutaneous adipose tissue; VAT = visceral adipose tissue, IHL = intra-hepatic lipids; MAT = marrow adipose tissue

Table 2 shows the results of regression analysis, revealing the association of MAT to IHL, HOMA-IR, leptin, and OC. However, after adjustment for age and weight, only the association of MAT to VAT (R^2^ = 0.44; p < 0.01, estimate = 0.00002) and IHL (R^2^ = 0.41; p < 0.05, estimate = 0.01) remained significant.

VAT and IHL showed a positive relationship with weight, HOMA-IR, and leptin and a negative relationship with adiponectin (Table 3). The regression analysis showed that the association remained significant after adjustment for VAT with IHL, HOMA-IR, and adiponectin (Table 2).

**Table 3:** Correlation of visceral adipose tissue and intra-hepatic lipids with weight, HOMA-IR, leptin and adiponectin.

Parameters	Weight	HOMA-IR	Leptin	Adiponectin
VAT	0.722*	0.643*	0.581*	-0.531*
IHL	0.579*	0.506*	0.362*	-0.412*

Note: HOMA-IR = Homeostatic model assessment—insulin resistance; VAT = visceral adipose tissue; IHL = intra-hepatic lipids

*p<0,05

## Discussion

The present study suggests a striking difference in the distribution of adipose tissue between CD and other individuals of similar body weight. CD not only accentuates the accumulation of fat in liver and visceral pad; it also increases bone marrow adiposity. MAT has a negative association with TBS and BMD. CD exacerbates several negative traits present in primary obesity, e.g., low 25-OHD, OC, and TBS, as well as high SAT, VAT, and IHL. Furthermore, CD changes the BMD and MAT profile, decreasing bone mass while expanding MAT.

Hypercortisolism causes well-known metabolic effects such as insulin resistance, increased gluconeogenesis, proteolysis, and lipid spillover. It has also been recognized as a fat-partitioning disorder that, in addition to insulin resistance, promotes susceptibility to severe metabolic disorders (14). Although the HOMA-IR mean values in the P group were higher than the threshold for the Brazilian population [24], CD individuals exhibited HOMA-IR significantly greater than the P group. In addition, the low adiponectin levels observed in CD favor increased morbidity and mortality regarding cardiometabolic risk.

Glucocorticoids are considered the main cause of secondary osteoporosis [25]. The present study assessed the spectrum of bone mass and TBS in CD patients compared to subjects with normal weight and a paired group. Regarding bone-related biochemical parameters, CD patients showed normal calcium, phosphorus, and PTH levels; higher alkaline phosphatase; and lower 25-OHD and osteocalcin levels. OC, a molecule originated in osteoblasts, acts on bone mineralization, a process also modulated by vitamin D. Altogether, the low circulatory levels of 25-OHD and OC, associated with low lumbar spine BMD and TBS, suggest a deeply negative effect of hypercortisolism on bone mass and bone quality in CD patients. Moreover, in CD, the increased storage of fat in both MAT and VAT, as well as the overflow of lipids to the liver, seem to negatively affect BMD and TBS. The same pattern is observed concerning the association of HOMA-IR to BMD and TBS in CD.

In accordance with previous studies, glucocorticoids typically have greater detrimental effects on trabecular than on cortical bone [26, 27]. The present results reflect this tenet, as the only site showing significant difference in BMD between the CD and P groups was the lumbar spine. However, with regard to the rate of BMD/BMI in femoral neck and total hip, the CD group showed lower values than the control group. This aspect is especially relevant in CD, as these subjects typically exhibit sarcopenic obesity [28], a condition associated with greater risk of bone fracture [29].

The TBS is considered a sensitive tool to detect bone abnormality in acromegaly and type 2 diabetes, conditions in which fracture susceptibility coexists with normal BMD [30, 31]. The present study supports previous studies showing that obesity [32] and Cushing’s syndrome [26] have detrimental effects on the TBS. Indeed, it has been optionally incorporated into the FRAX algorithm for estimating bone fracture risk [33, 34]. The present data show that CD patients exhibited lower TBS than primary obesity patients, indicating that TBS assessment has sufficient sensitivity to detect this spectrum of variation and it that may be useful to discriminate fracture risk in CD. It has to be highlighted that only the CD group included individuals with diabetes mellitus, but the TBS values were similar between the individuals with and without diabetes mellitus.

Hypercortisolism leads to a remarkable impairment in the differentiation and activity of osteoblast lineage cells, culminating in dysfunctional osteoblasts and increased apoptosis of osteocytes. Among the biochemical markers of bone remodeling, low circulating levels of OC are, as observed herein, a consistent sign of glucocorticoid excess. Szappanos and colleagues [35] proposed that OC measurements could be a biochemical parameter for the diagnosis of endogenous hypercortisolism. More interestingly, the present study showed a positive relationship between lumbar spine TBS and OC serum levels.

MAT was higher in CD patients compared to C and P subjects, with no difference between the two latter groups. The present results are in line with recent findings showing that MAT is not higher in the P group than in normal weight controls; this suggests that MAT is not a niche for lipid storage in a condition of energy surplus [13]. As such, MAT expansion does not line up with metabolic syndrome. Therefore, the present study contributes to this line of investigation, showing that CD individuals have higher MAT than those with primary obesity. However, we cannot exclude the direct effect of the hypercortisolism environment on bone marrow adipogenesis, as suggested in previous experimental studies [36].

The present study shows, for the first time, a negative relationship between MAT and TBS. In addition, TBS was negatively associated with VAT, indicating that the central obesity associated with CD may contribute to the impairment of bone quality in these patients. Our data are in agreement with previous studies showing that TBS was negatively affected by waist circumference [37] and trunk fat measured by DXA [38]. Since a negative association between TBS and VAT, HOMA-IR, and OC was observed, the present results provide indications that disorders in energy metabolism influence bone remodeling and bone quality in CD. It should be highlighted that the association remained after adjustment for body weight and age.

Although mice studies indicate that OC ameliorates insulin sensitivity [39], there are contradictory findings in human investigation [40]. However, the negative association of osteocalcin and HOMA-IR might suggest that low circulatory levels of OC may contribute to the insulin resistance in CD.

The present study has some limitations. Similarly to other studies in this line of investigation, the present study has a limited sample size, due to the rarity of CD. However, this study comprises only women, all having ACTH-dependent hypercortisolism due to pituitary adenoma, making the sample homogeneous. Additionally, the methods employed to assess adipose tissue measurements were the most appropriate for clinical investigation in this area, leading to a comprehensive study examining the impact of energy metabolism alterations on bone deterioration in CD.

In summary, the bone assessment by TBS was sufficiently sensitive to detect a spectrum of variation in bone texture among control, paired, and CD groups. Our data also suggest that, besides low levels of 25-OHD and OC, in CD patients insulin resistance and dysfunctional adipose tissue (i.e., high MAT and abdominal SAT and VAT) are active players on bone deterioration, confirmed by lower lumbar spine BMD and TBS. Thus, our findings point out an additional component of the already well-known complex mechanisms of osteoporosis associated with hypercortisolism.
